# Health-related quality of life in hereditary angioedema patients treated with subcutaneous C1 inhibitor (C1-INH) therapy in Canada

**DOI:** 10.1186/s13223-025-00991-2

**Published:** 2025-12-08

**Authors:** Susan Waserman, Gina Lacuesta, Amin S. Kanani, Gord L. Sussman, Harold Kim, Hugo Chapdelaine, Hadi A. Goubran Messiha, Chrystyna Kalicinsky, Adil Adatia, Belinda Yap, Marie-France Dansereau, Nataly Tanios, Rami El-Sayegh, Adriana Martin, Hachem Mahfouz, Maye Machnouk, Paul K. Keith

**Affiliations:** 1 Department of Medicine, McMaster University, Hamilton, ON Canada; 2 Dalhousie University, Halifax, NS Canada; 3 University of British Columbia, Vancouver, BC Canada; 4 Faculty of Medicine, University of Toronto, Toronto, ON Canada; 5 Division of Immunology, St. Michael’s Hospital, Toronto, ON Canada; 6 Division of Clinical Immunology and Allergy, Department of Medicine, Western University, London, ON Canada; 7 Clinique immunodéficience primaire de l’adulte, Institut de Recherches Cliniques de Montréal, Montréal, QC Canada; 8 Département de médecine, Centre hospitalier de l’université de Montréal, Université de Montréal, Montréal, QC Canada; 9 Saskatoon Cancer Center, Saskatoon, SK Canada; 10 Hematology Oncology, College of Medicine, University of Saskatchewan, Saskatoon, SK Canada; 11 University of Manitoba, Winnipeg, MB Canada; 12 Division of Pulmonary Medicine, Department of Medicine, University of Alberta, Edmonton, AB Canada; 13 Cencora, Innomar Strategies Inc, Oakville, ON Canada; 14 CSL Behring Canada Inc, Ottawa, ON Canada

**Keywords:** Hereditary angioedema, Health-related quality of life, SC-pdC1INH

## Abstract

**Background:**

Hereditary angioedema (HAE) therapies have demonstrated improvements in clinical symptoms and health-related quality of life (HR-QoL) in patients with HAE. Specific causes for improvement in HR-QoL have not been sufficiently explored in Canada.

**Objective:**

To understand patient perspectives on the burden of HAE and their experiences taking subcutaneous plasma-derived C1 inhibitor concentrate (SC-pdC1INH), and to investigate changes in HR-QoL domains which include physical, mental, emotional, functional, social, and medical well-being.

**Methods:**

A qualitative research design was employed using telephone interviews from May to July 2023 and a quantitative self-administered Angioedema QoL questionnaire (AE-QoL), which has a recall period of 4 weeks. A conceptual map was generated from the interviews that illustrated HR-QoL domains most important to patients.

**Results:**

This study included 20 patients (50% female; mean age of 51 years; 85% HAE Type 1/2). Patients reported more controlled HAE since starting SC-pdC1INH, with fewer attacks (85%) and even no/nearly no attacks (60%) in the past year. Patients reported emotional benefits (reduced stress/anxiety regarding potential attacks [75%], and increased confidence in managing HAE [95%]). Positive impacts also included increased productivity/less missed days from work/school (40%) and improved physical ability/well-being (50%). The total mean AE-QoL Score was 34 (range 0–87; SD 24.9), indicating a small effect of HAE on HR-QoL in the 4-week pre-interview period.

**Conclusion:**

Overall, patients on long-term prophylaxis with SC-pdC1INH reported improvements in many HR-QoL domains, but most significantly relief of stress and anxiety associated with HAE, and improvement in social and physical well-being.

## Introduction

Hereditary angioedema (HAE) due to C1-inhibitor deficiency is a rare autosomal-dominant disease,[1] with an estimated prevalence of 1:50,000 [2]. HAE is characterized by recurrent angioedema attacks most frequently affecting the face, limbs, upper airway, and abdomen [3]. Angioedema can cause debilitating symptoms, ranging from swelling of extremities to laryngeal edema and airway obstruction in extreme cases, which may cause mortality [4]. Recurrent angioedema substantially disrupts patient’s daily life through frequent absences from work or school, and decreased productivity [5]. HAE patients may experience stress and anxiety due to the chronic and unpredictable nature of attacks and ineffective treatment [4]. These can be a significant burden to patients and impair their health-related quality of life (HR-QoL),[ 6] with studies indicating poorer physical and mental health compared to the general population [7]. 

Treatment focuses on patients achieving a normal life by preventing and treating attacks, reducing morbidity and mortality, and improving HR-QoL [3, 8]. Recent advancements [3] include intravenous (IV) and subcutaneous (SC) formulations of plasma-derived C1-esterase inhibitor (pdC1-INH) being approved for routine prophylaxis to prevent HAE attacks in adolescents and adults. Although these therapies are highly effective, improving HR-QoL of HAE patients is an essential part of treatment success and disease management.

Many studies have shown improvements in HR-QoL from HAE therapies;[9–11] however, few have investigated the specific reasons behind improved HR-QoL scores. The purpose of this study is to conduct an in-depth, qualitative investigation of factors relevant to HR-QoL in the real-world among Canadian patients with HAE using SC-pdC1INH. Additionally, the qualitative research was supplemented by a quantitative assessment of patients’ QoL.

Specifically, this study examines the impact of SC-pdC1INH with two main objectives: (i) to understand the burden of HAE from the patients’ perspective and their experience using SC-pdC1INH; and (ii) to investigate patients’ perception of change in factors relevant to HR-QoL, including physical, mental, emotional, functional, social, and medical aspects in real-world patients using SC-pdC1INH.

## Methods

### Survey design

This study was conducted between May 4, 2023, to July 17, 2023, utilizing a semi-structured questionnaire (60-minute telephone interviews) and a validated Angioedema Quality of Life Questionnaire (AE-QoL). Interviews were based on an adapted version of the non-scripted semi-structured interview guide from Anderson et al.[3], to explore QoL concepts/domains in-depth (e.g., physical, mental, emotional, functional, social, and other QoL aspects). Interviews were recorded, with participant permission, using a secure phone system, ‘Rev^2^’,[12] to transcribe English audio files, which were deleted after verifying transcript accuracy. French audio files were transcribed manually. Recordings and transcriptions were anonymized.

The AE-QoL questionnaire was used to validate the qualitative results. This online, self-administered disease-specific tool was sent to patients before their 60-minute interviews. Participants answered questions regarding their QoL impairment in the 4-week period prior to interviews. Questions were grouped into four domains: functioning, fatigue/mood, fears/shame, and food, and individual raw scores were transformed into a linear scale (0-100). [3],[5],[13],[14] The questionnaire was hosted on ‘Qualtrics XM’ [15].

## Participants and recruitment

Participants were eligible for the study if they met the following criteria: HAE diagnosis, enrolled in CSL Behring’s patient support program (CSLB PLUS + PSP), treated with SC-pdC1INH for over 3 months, ≥ 18 years old, residing Canada, fluent in English or French, and able/willing to provide informed consent. Innomar Strategies and Bayshore HealthCare (CSLB PLUS + administrator) recruited eligible patients. Bayshore HealthCare had previously received consent to contact PSP patients, including for research purposes.

Participation in this study was voluntary and patients could decline participation at any point. Patient consent was electronically obtained prior to interviews and the AE-QoL survey. Nominal compensation was offered to all respondents, with compensation information anonymized and kept confidential. Innomar Strategies, as a third-party to the PSP and manufacturer, conducted interviews and had no prior relationship with the participants. The study was approved by the Health Ethics Research Board at Advarra.

### Data analysis

Thematic analysis was conducted on all interviews (*n* = 20), using methods based on grounded theory principle [16] to identify common themes and concepts within the data [3]. Two researchers reviewed the interview transcripts, developed the coding tree, and aggregated the data into categories, which were then presented to all authors for input on the thematic analysis/categorization. Concepts mentioned by at least a quarter of respondents (*n* = 5) were included in the conceptual model. Main themes were pre-determined based on prior research [3], the domains present in the validated HAE-QoL questionnaire, and the authors’ own clinical expertise with HAE patients. Positive changes as well as negative changes were included in the model.

The validated online AE-QoL questionnaire data was analyzed using ‘WINCROSS DESKTOP^®^20’,[17] which provided descriptive statistics. AE-QoL scores were calculated on Excel.

## Results

### Demographic characteristics

The study included 20 patients with HAE (124 patients met the eligibility criteria out of 400 enrolled in the PSP) (Table 1). Most patients were female (50.0%) versus 45.0% male and 5.0% non-binary. Most patients were 55–64 years old (40.0%) (mean age: 51 years [range: 24–84]). Half of the patients worked full-time, while 20% were unemployed or on disability. Most patients had HAE Type 1 (50.0%), followed by HAE Type 2 (35.0%) and Normal C1-INH (15.0%). All patients were prescribed SC-pdC1INH as routine prophylaxis, with most using it for 1–2 years (65%). In the past year, 35.0% of patients had 1–5 attacks, and 25.0% had 0 attacks, however, four patients (20.0%) reported > 15 attacks.

**Table 1:** Demographic characteristics of study population

	Respondents (*n* = 20)
Gender	
Male Female Non-Binary	9/20 (45.0%) 10/20 (50.0%) 1/20 (5.0%)
Age (years)	
18–24 25–34 35–44 45–54 55–64 65–74 75+ Mean	2/20 (10.0%) 1/20 (5.0%) 6/20 (30.0%) 0/20 (0.0%) 8/20 (40.0%) 0/20 (0.0%) 3/20 (15.0%) 51.0 (range: 24.0–84.0)
Province*	
Atlantic Canada Prairies (Alberta, Saskatchewan, Manitoba) Ontario BC	2/20 (10.0%) 8/20 (40.0%) 7/20 (35.0%) 3/20 (15.0%)
Employment Status	
Disability Unemployed Retired Part-time Full-time	1/20 (5.0%) 3/20 (15.0%) 3/20 (15.0%) 3/20 (15.0%) 10/20 (50.0%)
HAE Type	
Type 1 Type 2 Normal C1-INH	10/20 (50.0%) 7/20 (35.0%) 3/20 (15.0%)
Time since Diagnosis (years)	
0–5 6–10 11–20 21–40 > 40 Mean	5/20 (25.0%) 9/20 (45.0%) 2/20 (10.0%) 3/20 (15.0%) 1/20 (5.0%) 15.0
Time since using SC-pdC1INH (years)	
< 1 1–2 ≥ 3 Mean	2/20 (10%) 13/20 (65%) 5/20 (25%) 2.0
Prophylaxis Treatment Type Prior to SC-pdC1INH	
Patients on Prophylaxis prior to SC-pdC1INH IV-pdC1INH 500/1500 Cetirizine Danazol Lanadelumab	15/20 (75.0%) 9/15 (60.0%) 1/15 (7.0%) 3/15 (20.0%) 2/15 (13.0%)
On-Demand Treatment Types**	
IV pdC1INH 500/1500 Icatibant SC-pdC1INH (off-label use)	4/20 (20%) 13/20 (65%) 3/20 (15%)
Frequency of Attacks/Year	
0 1–5 6–10 11–14 15+	5/20 (25.0%) 7/20 (35.0%) 1/20 (5.0%) 3/20 (15.0%) 4/20 (20.0%)
Laryngeal attack or ER visits/hospitalizations	
Laryngeal attack (at any time) ER Visits (2–5 times in past year) Hospitalizations (1–3 in past year)	11/20 (55.0%) 6/20 (30.0%) 4/20 (20.0%)
Comorbidities	
Reported comorbidity Asthma Cancer (ovarian, prostate) Diabetes Hepatic steatosis Gout Cardiovascular disease ^∞^ Hypertension Irritable bowel syndrome MELAS syndrome Osteonecrosis Pernicious Anemia Polycythemia Rheumatoid Arthritis Seasonal allergies Urticaria^†^	12/20 (60.0%) 1/12 (8.0%) 3/12 (25.0%) 2/12 (17.0%) 1/12 (8.0%) 1/12 (8.0%) 2/12 (17.0%) 3/12 (25.0%) 1/12 (8.0%) 1/12 (8.0%) 1/12 (8.0%) 1/12 (8.0%) 1/12 (8.0%) 1/12 (8.0%) 2/12 (17.0%) 2/12 (17.0%)

*SC-pdC1INH is not available for patients in Quebec.

**All patients were being treated with SC-pdC1INH for prophylaxis for at least 3 months at the time of the study,* but the on-demand treatment for patients was either IV-pdC1INH or icatibant or off-label sc-pdc1inh.*

^∞^*Heart problems include: upper right carotid aneurysm*,* myocardial infarction*

*†Both patients with urticaria are HAE Type 2 on SC-pdC1INH for prophylaxis treatment and icatibant for on-demand treatment.*

### Impact of SC-pdC1INH: HR-QoL concepts

**Fig. 1:**
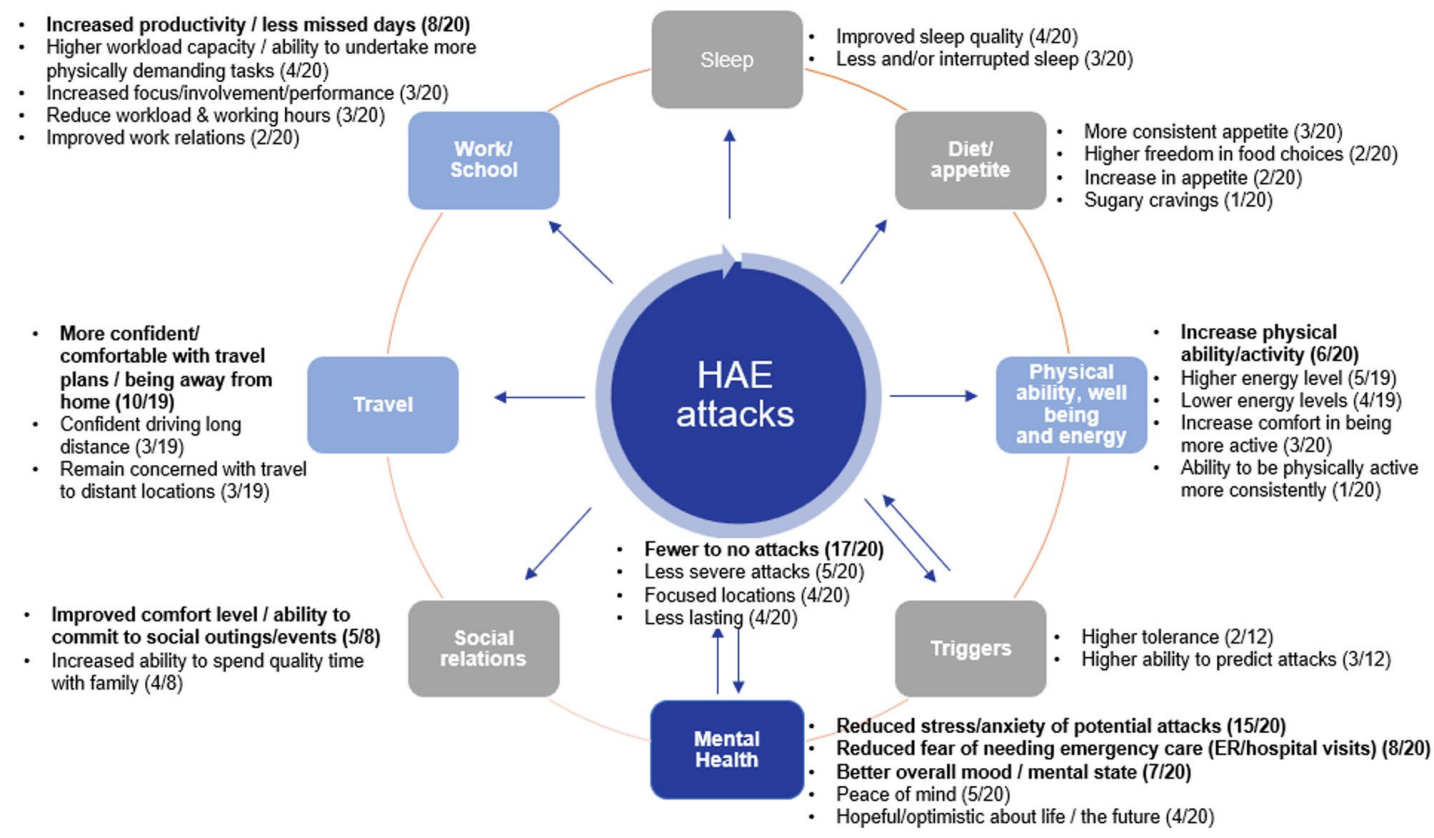
Thematic Analysis – HR-QoL conceptual model: impact of SC-pdC1INH

The analysis confirmed the 8 pre-identified themes on the impact of SC-pdC1INH, with no new themes emerging; however, new concepts within the existing themes were identified and grouped accordingly. The main themes included mental health, work/school, travel, diet/appetite, physical ability/well-being/energy, school relations, triggers, and sleep (Fig. 1). This model illustrates how SC-pdC1INH affects various areas that lead to improved QoL, largely due to a reduction in attack frequency.

## HAE attacks and frequency

Most patients reported better controlled HAE since starting SC-pdC1INH, reflected by several indicators. First, patients noticed a reduction in the number of attacks (17/20), with a quarter of patients (5/20) having no attacks in the previous year. When attacks did occur, some patients (4/20) reported shorter swelling episodes (2–3 days versus 7–10 days), and less severe and less painful attacks (5/20). Finally, some patients noticed slower spreading and greater confinement to certain areas, occurring in less painful locations (i.e., abdominal regions), or life-threating areas (i.e., throat, airways, etc.) (4/20). This theme is well summarized by one patient: “*Prior to [SC-pdC1INH] … if I had an abdominal attack*,* I would be laying up in bed for hours on end*,* days on end*,* doubled over in pain… now I find that they’re not quite as severe*,* they don’t last as long. I can tolerate things a lot better…My triggers still tend to be my triggers*,* but they have to be a little more extreme*.”

## Triggers

Among patients who had an attack since starting treatment and were familiar with their triggers, many reported no change in their triggers (7/12). Many other patients reported positive change (5/12) with greater capability at identifying and avoiding triggers, with some patients reporting a higher threshold for their known triggers (i.e., cold, stress, physical activity, certain foods, etc.) (4/12) and an increased ability to detect potential attacks when on SC-pdC1INH (3/12). However, some patients may not be able to identify the triggers for their attacks.

### Mental health and social relations

Patients shared emotions of pain, fear, anger, frustration, stress, anxiety, and feeling limited/restricted, largely due to the stress and anxiety of not knowing when the next attack might happen. Patients expressed that they especially had these feelings during the lengthy time between initial symptoms and HAE diagnosis and access to effective medication (8/20).

Notably, 85.0% (17/20) of interviewed patients reported a more positive outlook on living with HAE compared to the beginning of their HAE journey, with half (10/20) specifically crediting SC-pdC1INH for improvements in their HAE and overall QoL. Patients expressed that having the correct diagnosis and access to effective prophylaxis and on-demand therapies provided tremendous relief compared to living with uncertainty. This included reduced stress and anxiety about potential attacks (15/20), and less worry about being vulnerable and needing to rush to the ER/hospital (8/20). Patients reported increased optimism about life and living with HAE and hope for future generations’ access to effective therapies (5/20). Furthermore, patients felt more reassured that their condition was more predictable, and that they could rely on SC-pdC1INH and their on-demand therapies (5/20). Thus, patients reported a positive impact on their social relations; such that, better control of their HAE lead to increased participation in quality time with friends and family. They felt less apprehensive about making and keeping long-term and short-term plans and missing fewer events (5/8). As one patient stated: “*The anxiety*,* the fear level is gone… you don’t realize how much fear is there until it’s gone. You have a very different perception of the world and what you can do with your grandchildren or what you can do with your wife or your family*,* or friends*.”

### Travel

Patients felt more comfortable being away from home due to their ability to rely on themselves to detect a potential attack, to self-administer their emergency medication, and to recognize when to seek emergency care and explain their condition and treatment requirements. About half of the respondents (10/19) seemed more confident travelling, particularly due to their ability to rely on themselves to administer SC-pdC1INH to prevent attacks, especially among those who had seen a significant improvement in control since starting treatment (6/19). Patients also felt more experienced, more knowledgeable, and more capable of managing their disease while on SC-pdC1INH (5/19). One patient stated regarding being away from home: *I feel like I can control it now*,* there’s that sense of I don’t have to rely on other people to save me… I know that if there was a breakthrough despite the [prophylaxis]*,* that I would be okay [because of the on-demand treatment as back-up]”.*

However, some patients remained hesitant to be away from their local healthcare providers (3/19) or felt the need to plan and predetermine the location of the nearest hospital (2/19). Others found it problematic to travel with large quantities of medication requiring special storage requirements (4/19).

### Work/School

Before using SC-pdC1INH, patients mentioned having colleagues and/or bosses who were unfamiliar with HAE, leading to skepticism about frequent sick days or reduced involvement at work (e.g., missed business travels, avoidance of physically demanding/triggering tasks, amount of time away from work/schoolwork etc.) (2/20). Due to their condition, some patients had to adjust their workload, responsibilities/tasks and reduce working hours to limit any potential triggers (3/20). While other patients expressed no change in their work capacity since starting SC-pdC1INH, most noted missing fewer days due to their HAE (8/20) while being able to better focus and maintain their work involvement and performance levels since they were less ill/in less pain (3/20) and physically more capable/have a higher workload capacity (4/20). A couple of patients noted being able to consider more career opportunities (e.g., undertake more responsibilities) (2/20). One patient attributed their career to sc-pdC1INH prophylaxis treatment: *“I wouldn’t have this career path that I’m on right now without [sc-pdC1INH] because I just felt too unreliable…It’s just the pain is extraordinarily awful”.*

### Sleep, diet and appetite

Most patients noticed no change in their sleeping patterns (12/20). Those who noticed improved sleep attributed this to SC-pdC1INH’s ability to control their HAE which subsequently reduced their anxiety about waking with an attack (5/20). For example, one patient expressed not worrying about the dangers of going back to sleep: *“…if I wake up*,* I don’t think ‘is it safe to go back to sleep.’”*.

Some patients (3/20) did report sleeping less, however, attributed this to other factors like aging. Most patients reported no change in their diet and appetite (14/20), with some even reporting a positive change especially since being properly diagnosed and treated. One patient stated: *“…since being on [SC-pdC1INH] things as far as my appetite have not necessarily plateaued*,* but they’ve gone back to normal.”*

### Physical ability/well-being and energy levels

Half of patients (10/20) reported an overall improvement in their physical capacity allowing them to be more active due to being less sick and having an increased tolerance to physical triggers. In contrast, 8 patients (8/20) noticed no difference in their physical well-being and 2 patients reported feeling more tired and less inclined to be active, although they were unsure if this could be attributed to SC-pdC1INH or other factors such as lifestyle changes or age. Patients reported being able to go on longer walks, hikes, bike rides, stand for longer, and bend over without swelling, allowing them to pursue and consider activities that were previously off-limits prior to SC-pdC1INH (6/20). Some patients also expressed having less fear of swelling or attacks while doing physical activities (2/20).

Regarding energy levels, over half of patients reported no change (10/19). Some patients (4/19) noted a decrease in their energy level for unknown reasons, though one of these patients attributed their lack of energy to a recent HAE attack. Other patients felt more energetic, reporting increased productivity, having more energy for physical activity, household chores, and play with their children (5/19).

### Perceptions of SC-pdC1INH and managing HAE

Since starting on SC-pdC1INH, nearly every patient (19/20) reported increased confidence and comfort managing HAE. Overall, SC-pdC1INH had a mostly positive impact or no impact on the evaluated elements affecting patients’ QoL. SC-pdC1INH was reported to have a more positive rather than negative impact on various HR-QoL aspects with 25% to 95% of patients reported having some level of improvement compared to only 5% to 21% who perceived a deterioration in some evaluated HR-QoL areas. The biggest positive impact reported related to confidence and comfort in managing HAE (19/20), HAE attacks (19/20), and stress/anxiety levels (17/20) (Fig. 6). The least impact was observed in patients’ diet/appetite, energy level, sleep, driving comfort and long-term planning, with 57% to 70% of patients reporting no impact.

### AE-QoL validated survey: impact of HAE on patients’ daily lives (4 weeks prior to study)

**Fig. 2:**
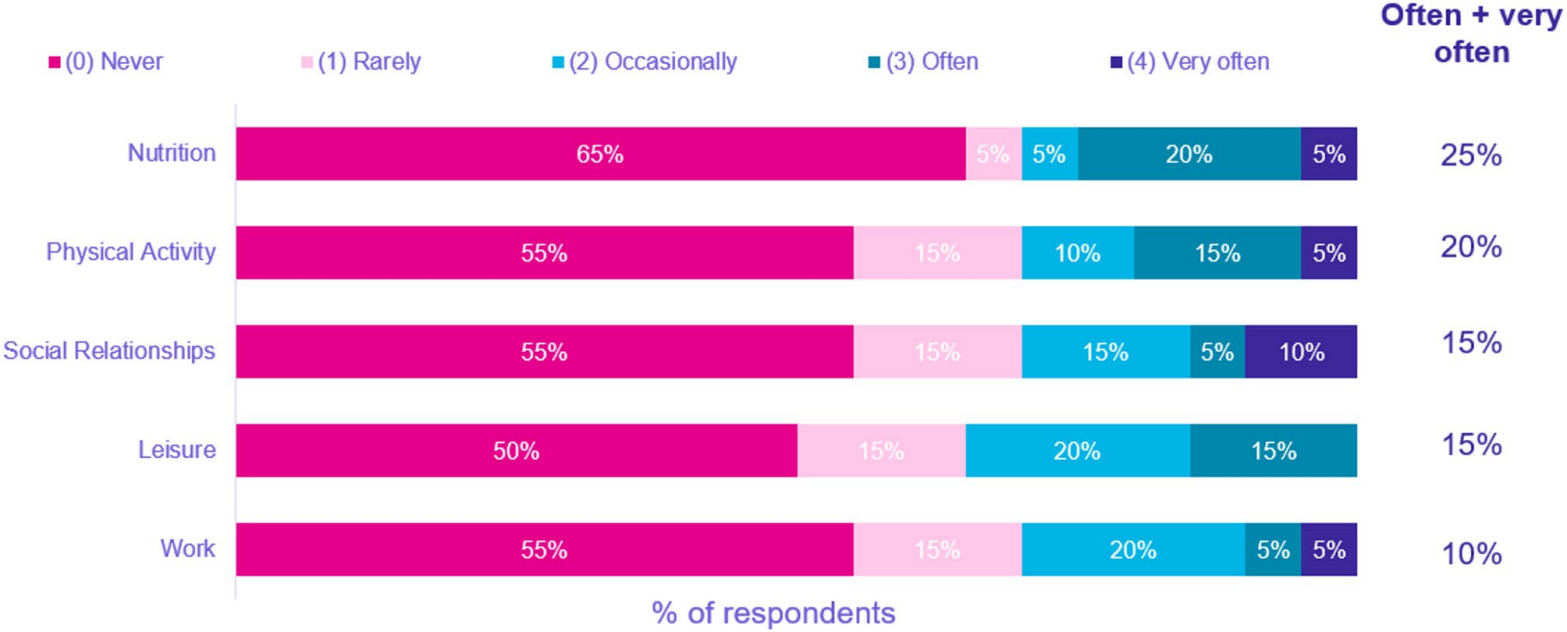
How often HAE impacts patients’ daily lives: measured by AE-QoL survey

**Fig. 3:**
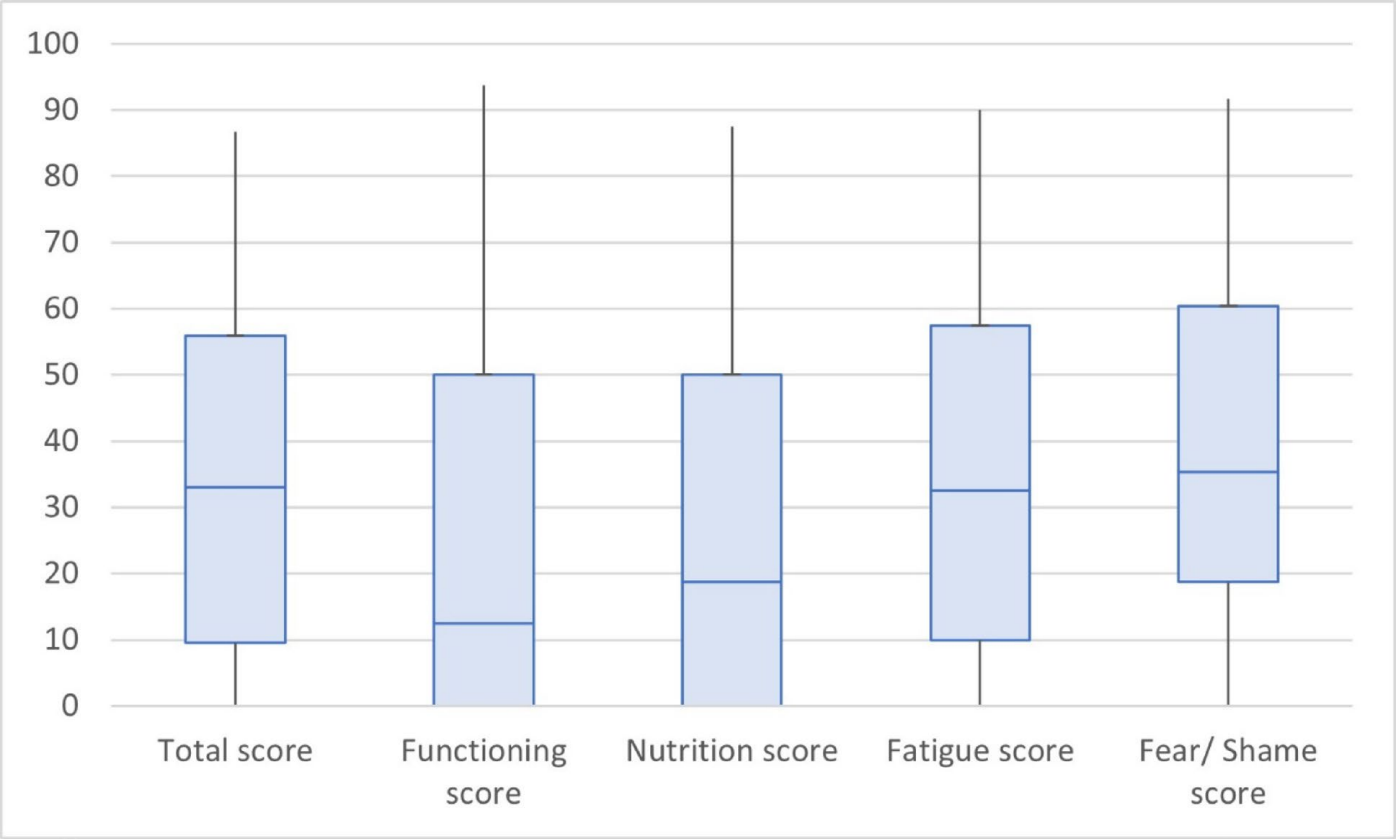
Median and interquartile range of each AE-QoL domain

**Fig. 4:**
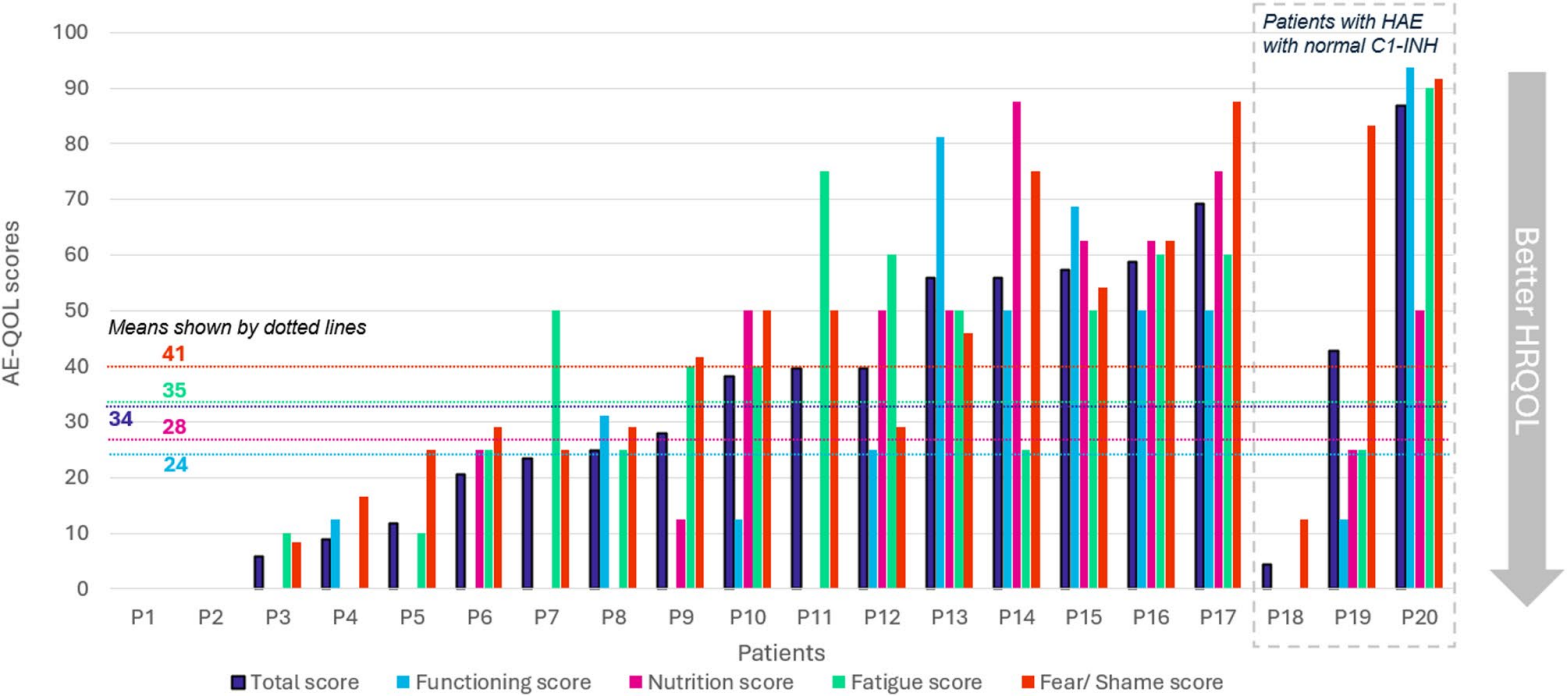
Individual AE-QOL scores per domain: 4 weeks prior to study

The questionnaire reflected disease specific QoL in the 4 weeks prior to interviews. Notably, at least half of all 20 patients selected ‘never’ being impacted by HAE in this pre-interview period across the domains of nutrition, physical activity, social relationships, leisure, and work (Fig. 2). The total AE-QoL Score had a median of 33.1 (IQR 9.6–55.9). HAE had the biggest impact on fear and shame (median 35.4, IQR 18.8–60.4), followed by fatigue (median 32.5, IQR 10.0-57.5), nutrition (median 18.8, IQR 0–50.0), and functioning (median 12.5, IQR 0–50.0 (Fig. 3). The total mean AE-QoL Score was 34 (range 0–87; SD 24.9), indicating a wide range of HR-QoL scores due to HAE (Fig. 4).

**Fig. 5:**
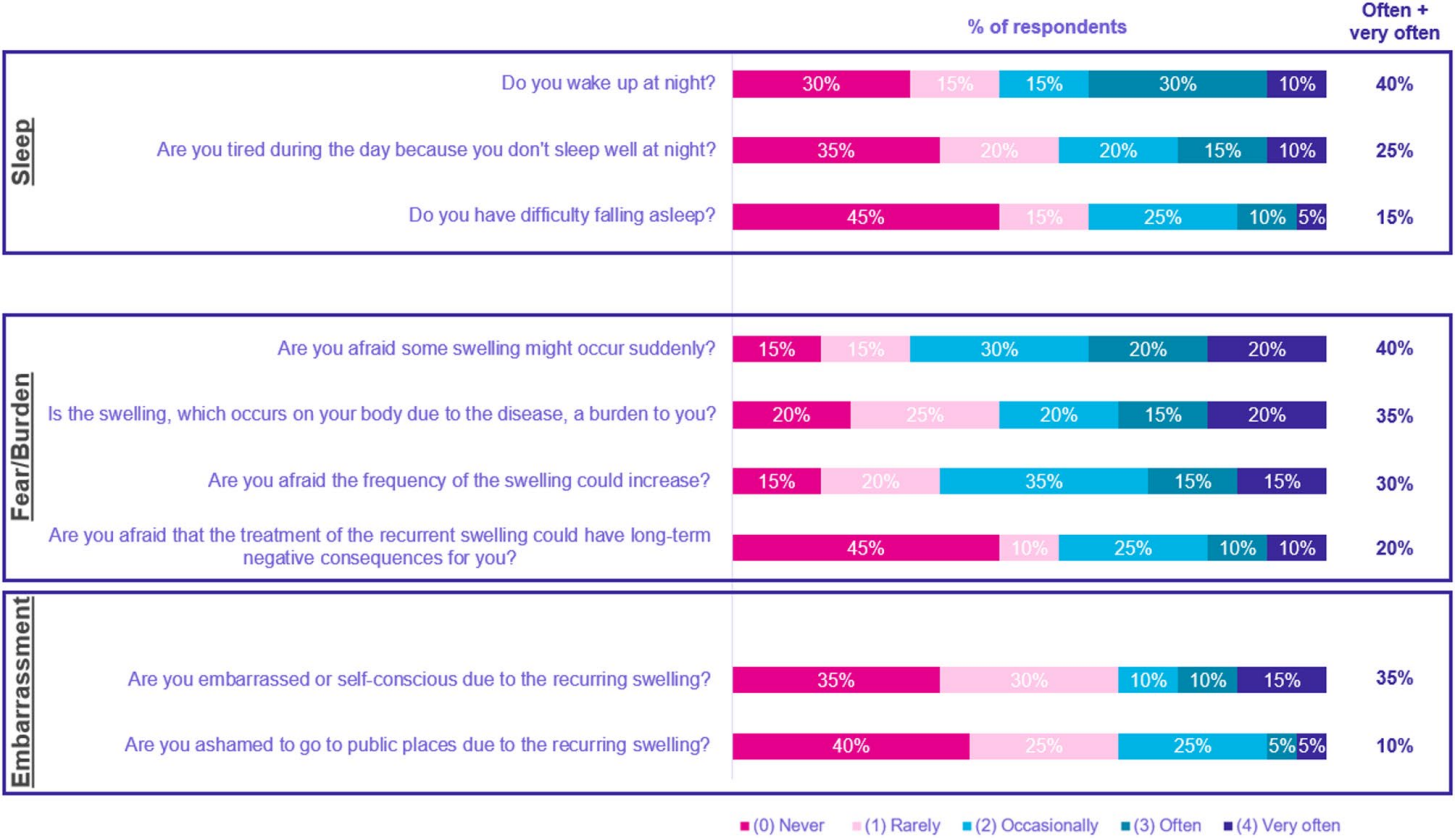
Impact of HAE on patients’ daily lives: effects on sleep, fear, and embarrassment of attacks and swelling

Regarding sleep, nearly 40% of patients reported sleep issues, and patients reported ‘*often/very often’* waking up at night (40%) as the most common reason, followed by daytime tiredness because of poor sleep (25%). Fear of attacks and swelling were shown to be a concern for many patients as well. This included patients reporting ‘*often/very often’* having a fear of sudden swelling (40%), feeling burdened by the swelling (35%), being afraid of the swelling increasing in frequency (30.0%), and embarrassment or self-consciousness associated with swelling reported by 25% as *‘often/very often’* (Fig. 5).

**Fig. 6:**
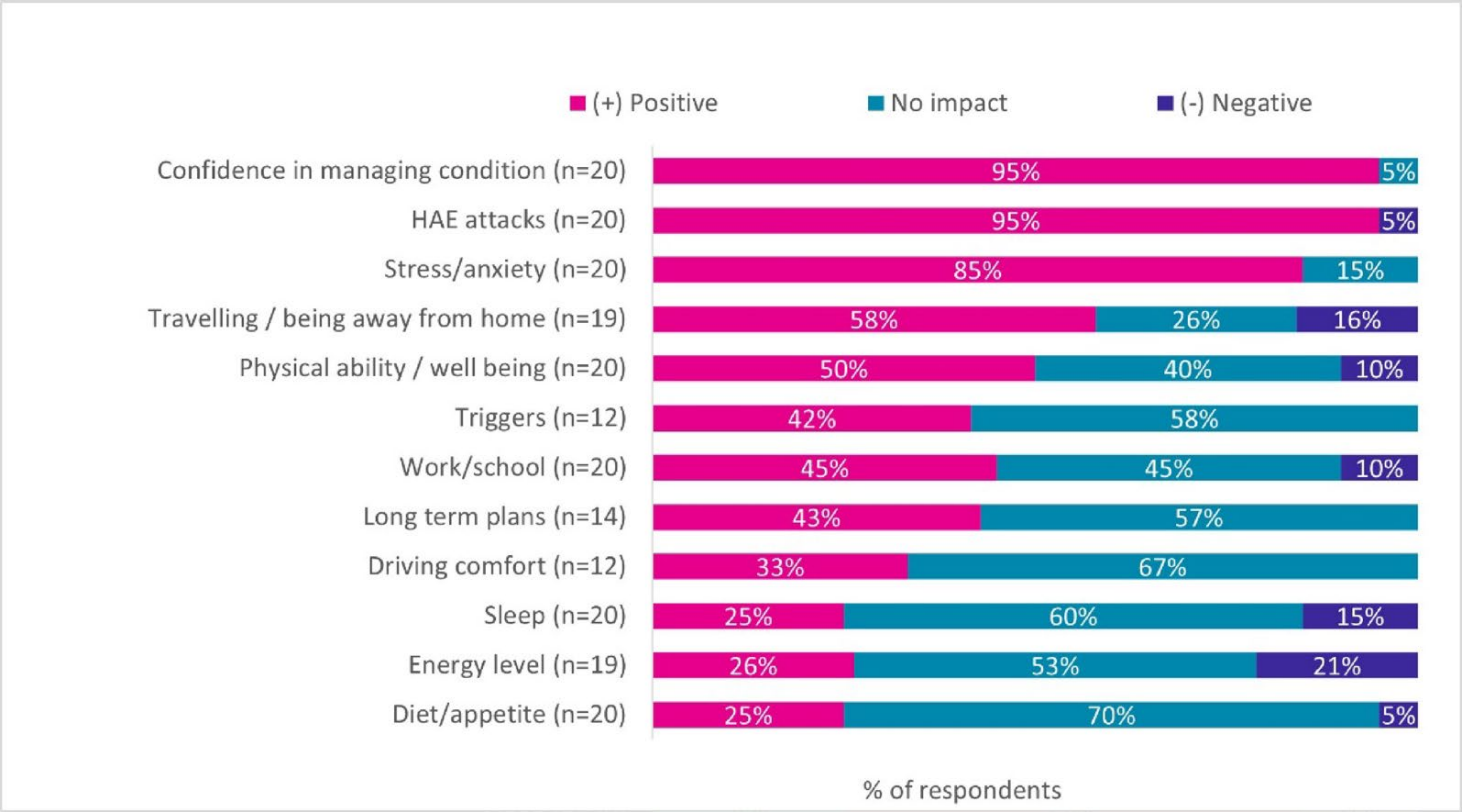
Overall summary of impact of SC-pdC1INH

## Discussion

The interviews revealed real-world experiences of patients since starting SC-pdC1INH, mapping key concepts/themes that patients expressed as benefits to their overall HR-QoL. HAE significantly impacts their lives, as they reported an inability to lead a ‘normal’ life due to stress and anxiety related to the unpredictability of attacks. They described often missing family and friend gatherings, struggling to thrive at work, avoiding travel, and avoiding physical activities, further exacerbating their stress and anxiety. The conceptual model shows the interconnectedness of the HR-QoL domains. For instance, nearly all patients in this study reported improvements in their overall health since starting SC-pdC1INH. They reported reductions in attack frequency and severity, along with greater awareness and a higher threshold for attack triggers. This seemed to positively influence stress and anxiety, and thus enhanced professional, social, and physical health. Patients reported experiencing greater freedom from their condition, which fosters more confidence in their daily lives, with fewer limitations.

The AE-QoL questionnaire was employed to validate the experiences shared by patients in their interviews and to help them express HAE HR-QoL impacts that they might be less comfortable sharing with the interviewer. Overall, the results showed that in the 4 weeks prior to being interviewed, HAE did not have a frequent impact on their lives regarding work, nutrition, leisure, social relations, physical activity, and mental health, which is consistent with improvements expressed by patients in interviews. The mean AE-QoL score (34) indicated a small effect of HAE on HR-QoL in the 4-week pre-interview period; however, the scores showed poorer scores in fatigue and fear/shame domains. These results indicate that while patients expressed in their interviews improvements in anxiety compared to before starting pd-C1INH, the fear of having an attack still affects over a third of patients. Such emotions have significant implications, leading to limitations in workplace participation and avoidance of physical activities, travel, and social interactions. Therefore, this study suggests that patients would benefit from quicker diagnosis and access to appropriate treatment to limit the impacts on QoL, particularly stress/anxiety/fear that may linger after treatment initiation.

The AE-QoL survey had a wide range of scores, with the median score comparable to patients having 1–2 attacks per month in a long-term extension study of patients on SC-pdC1INH (mean score of 34.4 at Week 70) [18]. Some patients had scores that were similar to those on no prophylaxis/placebo (mean score of 47.1).^18^ Three patients indicated excellent control (scores of less than 12). The variability could be attributed to the possibility that some patients were not taking an optimal therapeutic dose (60 u/kg twice weekly), potentially leading to a sub-optimal response (medical records were not available to confirm patient dosing). Regardless, patient feedback in interviews reflected a positive experience since starting SC-pdC1INH. Almost all patients perceived SC-pdC1INH as an effective and well-tolerated treatment that improved predictability regarding disease control, notwithstanding, the improvements in the other domains analyzed in the thematic analysis.

This study presented similar themes as two qualitative US-based studies on patient perspectives of impact of SC-pdC1INH on HR-QoL,[3,19] with similar positive impacts as those reported in the Anderson et al. study [3]. This study adds to the literature a deeper understanding of the pre-identified themes, while also uncovering new categories/sub-categories from the patient-reported data. For instance, this study additionally validates the results of the questionnaire by employing a survey that uses a quantitative design. Specific aspects of patient experiences are highlighted that that can be targeted for further improvements in HR-QoL, such as, managing patient stress, anxiety, and fears, or sleep and fatigue that may persist after improved HAE after initiation of SC-pdC1INH.

While baseline values were not collected prior to therapy, patients were asked to recall their situation before starting SC-pdC1INH. Future research would benefit from a longitudinal study design to assess change in QoL, including identifying characteristics that predict improved/worsened QoL outcomes. This study, through the interviews, tried to understand which specific QoL concepts improved or still lagged since starting SC-pdC1INH. The patient experiences expressed in this study contribute to existing literature showing improvements in HR-QoL, including long-term improvements in overall health status, anxiety, work and social impairment versus placebo (e.g., as seen in a long-term open-label extension study, post-hoc analysis, and a literature review) [10, 18, 20]. 

Potential limitations align with those commonly encountered in survey studies. The reliance on patient-reported data may have introduced recall bias, as respondents might struggle to remember details about their HAE experience (e.g., average weekly doses, HAE type, and treatment type). Furthermore, this study only had 20 patients, potentially causing some outliers to skew the data, especially in the AE-QoL study. There is also possibility of selection bias since patients were representative of the CSLB PLUS + PSP and may not reflect the broader HAE population. However, this sample size is typical in studies for rare diseases, including HAE [3, 21, 22]. Also, given the variability in patient experiences,[23–27] and considering the results are consistent with conclusions of previous studies,[3,10,18–20] it is reasonable to assume that the findings of this study are similar to the experiences of HAE patients population on SC-pdC1INH in Canada. Furthermore, meaning saturation of certain conceptual categories was achieved after 14 interviews when no new sub-categories or new meanings emerged, especially positive improvements on HAE attacks, stress/fear/anxiety, comfort and confidence in managing HAE, travel, and limited impact on diet/appetite.

HAE patients in this study reported severely impaired QoL prior to sc-pdC1INH treatment, and overall, sc-pdC1INH improved their HR-QoL by reducing HAE attacks, frequency, and severity, which subsequently reduced stress, anxiety, and overall limitations of living with HAE. It is critical to increase awareness among physicians about HAE diagnosis and its symptoms to ensure that patients receive treatment as soon as possible, to limit HAE’s impact on HR-QoL. Given the significant burden of HAE on QoL, this study supports the need for longitudinal research to assess the impact of HAE on patient HR-QoL and the extent of improvements experienced during treatment.

## Data Availability

The data that support the findings of this study are not openly available due to reasons of sensitivity and are available from the corresponding author upon reasonable request, but restrictions apply to the availability of these data.

## Abberivations

AE-QoL: Angioedema Quality of Life Questionnaire
C1-INH: C1 esterase inhibitor
HAE: Hereditary angioedema
HR-QoL: Health-related quality of life
IV: Intravenous
REB: Research Ethics Board
SC: Subcutaneous

## Declarations

**GL** has received consultancy and/or speaker fees from Takeda, CSL Behring, BioCryst, KalVista, and is a member of CHAEN;

**ASK** received honoraria from CSL Behring, Takeda, BioCryst;

**GLS** is a medical advisor for Novartis Pharmaceuticals and CSL Behring; has also been contracted for research by Amgen, Aimmune Therapeutics, AstraZeneca, DBV Technologies, Leo Pharma, Novartis Pharmaceuticals, Sanofi, Regeneron Pharmaceuticals, Innovaderm Research Inc., Escient Pharmaceuticals, CSL Behring, Kalvista Pharma, Pharvaris and Aslan Pharmaceuticals;

**HK** has been a medical lecturer and/or has participated in advisory boards for ALK-Abelló, AstraZeneca, Bausch Health, CSL Behring, GSK, Miravo, Novartis, Medexus, Pfizer, Sanofi, Takeda, Valeopharma; is an Associate Professor at Western University and an Assistant Clinical Professor at McMaster University; serves as Editor-in-Chief of Allergy, Asthma and Clinical Immunology;

**HC** has been contracted for research by Sobi, Alexion, Sanofi, Takeda, CSL-Behring, Kalvista, Pharvaris, has received consultancy and/or speaker fees from Takeda, CSL Behring, BioCryst, Novartis, Pharming, and is a board member of CHAEN;

**HAGM** participated in advisory board for CSL Behring;

**CK** has received speaker/consultancy fees from CSL Behring and Takeda;

**AA** reports consulting fees and honoraria from BioCryst, Covis Pharma, CSL-Behring, Intellia, Kalvista, Pharvaris, and Takeda and clinical trial support from Astria, BioCryst, Intellia, Ionis Pharmaceuticals, Kalvista, Octapharma, and Pharvaris, outside of the submitted work;

**BY, MFD, NT,** and** RES **are employees of Cencora, Innomar Strategies, Inc;

**MM, HM,** and** AM** are employees of CSL Behring Canada Inc., Ottawa, ON, Canada;

**PKK** has been a speaker, advisory board member, or consultant for and has received honoraria from ALK- Abelló, AstraZeneca, Bausch, Bayer, BioCryst Pharmaceuticals, Canadian Agency for Drugs and Technologies in Health (CADTH), Canadian Pharmacists Association (CPA), CSL Behring, GSK, Medexus Pharmaceuticals Inc., Merck & Co., Novartis, Sanofi Genzyme, Takeda/Shire, and Valeo; has received research grants from CSL Behring and Takeda/Shire; serves as a medical advisor (volunteer) for Hereditary Angioedema Canada, a patient organisation; serves as a board member (volunteer) for the CHAEN, a physician organisation; and is an employee of McMaster University.

**BY, MFD, NT,**and** RES **analysis and interpretation of patient data and were major contributors in writing of the manuscript.

All authors read and approved the final manuscript.
